# Early detection and therapeutics

**DOI:** 10.1002/1878-0261.12458

**Published:** 2019-02-20

**Authors:** Wladyslaw Januszewicz, Rebecca C. Fitzgerald

**Affiliations:** ^1^ MRC Cancer Unit University of Cambridge UK; ^2^ Department of Gastroenterology, Hepatology and Clinical Oncology Medical Centre for Postgraduate Education Warsaw Poland

**Keywords:** cancer screening, colorectal cancer, endoscopy, gastric cancer, oesophageal cancer, preneoplastic conditions

## Abstract

Early detection, including cancer screening and surveillance, is emerging as one of the most important topics in modern oncology. Because symptomatic presentation remains the predominant route to cancer diagnosis, there is a growing interest in developing techniques to detect the disease at an early, curative stage. Moreover, growing understanding of cancer biology has paved the way for prevention studies with the focus on therapeutic interventions for premalignant conditions. Where there is a recognisable precursor stage, such as a colorectal adenoma or Barrett's metaplasia, the removal of abnormal tissue prevents the development of cancer and enables stratification of the patient to a high‐risk group requiring further surveillance. Here, we provide a review of the available technologies for early diagnosis and minimally‐invasive treatment.

**Abbreviations**
APC: argon plasma coagulation
CI: confidence interval
CRC: colorectal cancer; ctDNA; circulating tumour DNA
FAP: familial adenomatous polyposis
FIT: faecal immunochemical test
FOBT: faecal occult blood test
GI: gastrointestinal
GORD: gastro‐oesophageal reflux disease
HDGCS: hereditary diffuse gastric cancer syndrome
HGD: high‐grade dysplasia
IEN: intraepithelial neoplasia
IQR: interquartile range
LGD: low‐grade dysplasia
OAC: oesophageal adenocarcinoma
OSCC: oesophageal squamous cell cancer
RCT: randomised controlled trial
RFA: radiofrequency ablation
TFF‐3: trefoil‐factor 3
TNE: trans‐nasal endoscopy
VCE: video capsule endoscopy
VOC: volatile organic compound

## 1 Introduction

Early detection, including cancer screening and surveillance, is emerging as one of the most important topics in modern oncology. Because symptomatic presentation remains the predominant route to cancer diagnosis, there is a growing interest in developing techniques to detect the disease at an early, curative stage. Moreover, a growing understanding of cancer biology has paved the way for prevention studies with the focus on therapeutic interventions for premalignant conditions. Where there is a recognisable precursor stage, such as a colorectal adenoma or Barrett's metaplasia, the removal of abnormal tissue prevents the development of cancer and enables stratification of the patient to a high‐risk group requiring further surveillance.

The two main ways of screening for early neoplasia include imaging and molecular biomarker testing. Gastrointestinal (GI) malignancies, including oesophageal, stomach and bowel cancer, represent a group of diseases where both of these approaches have been extensively investigated. On the one hand, imaging for GI malignancy is tractable given the accessibility of the GI tract for imaging modalities including endoscopy. On the other hand, the high prevalence of GI cancers raises the need for less invasive and more cost‐effective methods and some molecular biomarkers have the potential to replace endoscopy as a screening modality. The utility of biomarkers expands beyond the primary detection to include monitoring of the course of the disease, response to therapy and early relapse detection after treatment. In this review, we have used the GI tract as an example to present the established and emerging screening methodologies for early cancer detection and highlight the minimally‐invasive treatment options that are radically altering the rationale and health economics for early cancer detection.

## 2 Technologies for early diagnosis

### 2.1 Endoscopy

#### 2.1.1 Conventional endoscopy

Endoscopy with biopsies remain the gold standard for the diagnosis of GI malignancies; however, its utility as a screening modality is limited to colorectal cancer (CRC). In 2015, 24 out of 28 countries in the European Union had established or upcoming country‐wide CRC screening programmes targeting the average‐risk population, defined as individuals aged 50 years or older with no additional risk factors (Navarro *et al*., 2017). Despite the well‐defined target population, there is a wide variation in screening practices among those countries resulting from different financial resources and healthcare system capacities. The recommended strategies include an annual or biennial faecal immunochemical test (FIT), sigmoidoscopy every 5 years or colonoscopy every 10 years. Endoscopic screening has the advantage of facilitating CRC prevention by removal of the precursor lesion (ie, colorectal adenoma) at the time of the initial examination. The first evidence suggesting that sigmoidoscopy is effective in CRC screening, with benefits lasting for up to ten years, comes from two case‐controlled studies performed in the early 1990s (Newcomb *et al*., 1992; Selby *et al*., 1992). Further evidence from a multicentre randomised controlled trial (RCT) showed that a single flexible sigmoidoscopy, performed in asymptomatic individuals aged 55–64 years, can reduce CRC incidence and mortality by 23% [hazard ratio = 0.77, 95% confidence interval (CI) = 0.70–0.84] and 31% (hazard ratio = 0.69, 95% CI = 0.59–0.82), respectively (Atkin *et al*., 2010). Sigmoidoscopy is a less invasive test compared to colonoscopy; however, it only examines the lower part of the large bowel, whereas colonoscopy can visualise the entire colon. This is particularly important in the light of evidence for a proximal shift in the distribution of CRC (de Oliveira *et al*., 2015). Therefore, colonoscopy is currently the preferred CRC screening test in the US (Klabunde *et al*., 2011) and several European countries (Zavoral *et al*., 2009), although there is no high‐quality data to support the effectiveness of colonoscopy and its superiority over other screening modalities. Three long‐term RCTs are currently underway and the results are highly awaited (expected in 2020 and beyond). These trials include the COLONPREV trial in Spain, comprising a non‐inferiority trial comparing biennial FIT vs. one‐time colonoscopy (Quintero *et al*., 2012); the CONFIRM trial in the US (ClinicalTrials.gov NCT01239082) comparing annual FIT vs. one‐time colonoscopy; and the NordICC trial in Northern and Eastern Europe (Kaminski *et al*., 2012), a randomised trial comparing once‐only colonoscopy screening with no screening.

**Figure 1:**
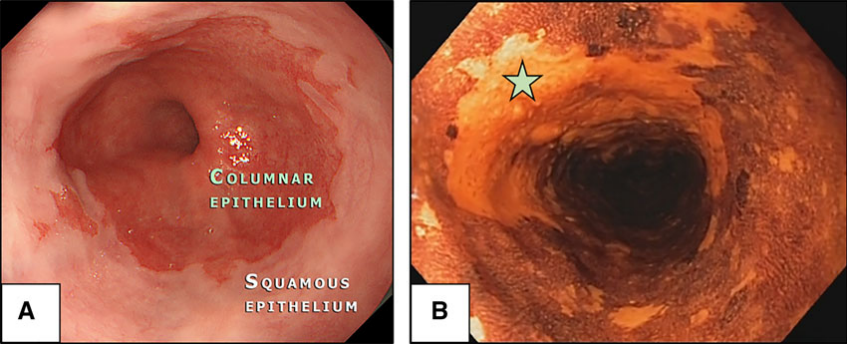
Barrett's oesophagus and squamous cell cancer of the oesophagus. (A) Endoscopic image of Barrett's oesophagus visible as a salmon‐coloured metaplastic epithelium (columnar) replacing the normal bright‐pink epithelium of the distal oesophagus (squamous). (B) Endoscopic image of an early squamous cell carcinoma in the oesophagus visible as an unstained area after Lugol's iodine staining (asterisk). Patients provided their written consent for the images to be used for educational purposes.

Oesophageal cancers, including oesophageal adenocarcinoma (OAC) and oesophageal squamous cell cancer (OSCC), have a relatively low incidence rate in the Western world and, as a result, general endoscopic screening has not been established. However, the rapidly increasing incidence of OAC in recent decades has been raised as a public health concern, especially in the UK, where the incidence and mortality for this cancer are the highest in Europe (Coleman *et al*., 2018). OAC is associated with obesity and gastro‐oesophageal reflux disease (GORD) and has a well‐established precursor condition, namely Barrett's oesophagus, which is defined as an endoscopically visible segment (≥1 cm) of metaplastic columnar epithelium in the distal oesophagus (Fitzgerald *et al*., 2014) (Fig. 1A). Although most individuals with Barrett's do not progress to adenocarcinoma, the progression rate is 0.3% per annum and the abysmal survival rate for OAC (unless detected at an early stage) justifies early detection efforts (Desai *et al*., 2012). Therefore, current guidelines from the British Society of Gastroenterology recommend a targeted screening approach, which involves a high‐definition white light endoscopy examination in high‐risk individuals. This is defined as patients with chronic GORD symptoms and at least three of other risk factors: age 50 years or older, white race, male sex and obesity (this threshold can be lowered for patients who have a first‐degree relative with Barrett's oesophagus or OAC) (Fitzgerald *et al*., 2014). This guideline is partly predicated on the feasibility and affordability of large‐scale endoscopy given the low prevalence of the condition estimated at around 2–3% of the population with reflux symptoms (Ronkainen *et al*., 2005).

OSCC has a wide geographical variation, with incidence rates up to 21.62 cases per 100 000 population in certain high‐incidence areas of China (Zeng *et al*., 2016), Korea or Iran, where population‐based screening is a viable option. In Western countries, however, the incidence of OSCC is low and continues to decline and therefore, general screening is not recommended (Smyth *et al*., 2017), with only high‐risk individuals such as patients after curative treatment for head and neck cancer, previous endoscopic resection of OSCC, caustic injury, tylosis and achalasia benefiting from endoscopic screening. By contrast to Barrett's oesophagus, squamous intraepithelial neoplasia (IEN), the precursor lesion for OSCC, can be invisible on standard white‐light imaging. Many advanced imaging techniques are available to highlight areas with IEN, although the simplest and most effective is Lugol's iodine staining (Codipilly *et al*., 2018). Dysplastic areas appear unstained given the absence of glycogen in the neoplastic cells (Fig. 1B) and several studies have shown that Lugol's dye staining increases the sensitivity for the detection of high‐grade IEN and early squamous cancer (Dubuc *et al*., 2006; di Pietro *et al*., 2018).

Screening for gastric cancer is widely available in countries with a high prevalence, such as China, Japan and Korea. In Japan, for example, the initial screening programme started in the 1960s and was based on photofluorography, which was offered to all residents aged 40 years and older. With time, endoscopy has become the investigation of choice for mass screening including several major Japanese cities. Similar to other GI malignancies, a non‐cardia gastric cancer commonly develops through a number of premalignant stages from chronic atrophic gastritis, by way of intestinal metaplasia, to dysplasia and cancer. This sequence, often triggered by Helicobacter pylori infection, is known as Correa's cascade (Correa *et al*., 1975). Endoscopy gives the opportunity to identify both the premalignant stage and early neoplasia. Although general screening for gastric cancer is not justified in Western countries, patients with previously recognised precancerous conditions require long‐term endoscopic surveillance (Dinis‐Ribeiro *et al*., 2012). The European guidelines recommend 3‐yearly monitoring for extensive atrophy and/or intestinal metaplasia in the stomach (involving both the antrum and the gastric body). Patients with dysplasia without an endoscopically visible lesion should be closely followed up, either immediately and 6 to 12 months thereafter, or within 12 months, respectively, for those with high grade or low‐grade dysplasia (Dinis‐Ribeiro *et al*., 2012). However clinically useful, these recommendations are based mainly on expert opinions and not on randomised controlled trials (Dinis‐Ribeiro *et al*., 2012).

Gastric cancer is usually sporadic, although 1–3% of these neoplasms arise on a background of inherited cancer predisposition (Hansford *et al*., 2015). This includes hereditary diffuse gastric cancer syndrome (HDGCS) associated with a germline mutation in the E‐cadherin gene (CDH1). Carriers of this mutation have a lifetime risk of gastric cancer reaching up to 80% (van der Post *et al*., 2015). Female carriers additionally have a risk of a lobular subtype of breast cancer ranging between 39% and 52% (Hansford *et al*., 2015). Individuals with a confirmed CDH1 mutation are recommended to undergo prophylactic gastrectomy; however, endoscopic surveillance may be offered to those opting not to have gastrectomy at the current time, with CDH1 mutation variants of uncertain significance and those that fulfill HDGC clinical criteria without germline CDH1 mutations. Such surveillance should be performed in experienced centres, with the use of high‐definition endoscopy equipped with advanced imaging modalities within a dedicated session of at least 30 min. Target biopsies from suspicious areas (pale in appearance) and multiple random biopsies from each segment of the stomach should be taken (more than 30 samples per session) (van der Post *et al*., 2015).

Similar surveillance programmes are available for patients with genetic syndromes predisposing to colorectal cancer, such as Lynch syndrome (mutation in one of the DNA mismatch repair genes) and familial adenomatous polyposis (FAP; mutation in the APC gene). For the former, the British guidelines recommend a biennial colonic surveillance regime starting at the age of 25 years (or 5 years less than the first cancer case in the family, whichever is the earlier) and biennial upper GI endoscopy in families where there are cases of gastric cancer, commenced at age 50 years (Dunlop, 2002). In FAP with documented APC gene mutations, surveillance might be offered as a temporary measure for those who wish to postpone the prophylactic colectomy for personal reasons. In these cases, six‐monthly flexible sigmoidoscopy and annual colonoscopy can be an option, although surgery should be strongly recommended before the age of 25 years. Following colectomy, the rectum must be kept under review at least annually, as well as the anorectal cuff after restorative proctocolectomy (Dunlop, 2002). Upper GI endoscopy should also be undertaken in FAP to identify adenomatous polyps and early cancers, usually within the duodenum. A 3‐yearly upper GI endoscopy is recommended from the age of 30 years (Dunlop, 2002).

Overall, endoscopy is characterised by high accuracy in cancer detection; however, it is an operator‐dependent procedure, with a miss rate of 9.4–11.3% upper GI cancers (Menon and Trudgill, 2014; Pimenta‐Melo *et al*., 2016) and 1.8–9.0% CRCs (Singh *et al*., 2014). Other downsides include invasiveness and high costs. Therefore, several alternative screening modalities are being proposed to overcome those limitations.

#### 2.1.2 Transnasal endoscopy

For oesophagogastric screening, ultrathin transnasal endoscopy (TNE) may be a promising alternative to standard endoscopy. As a result of its small diameter of only 6 mm, TNE is characterised by high tolerability and improved cost‐effectiveness (mainly because it does not require sedation). In previous studies, TNE was preferred by 59–71% of patients compared to standard endoscopy (Jobe *et al*., 2006; Shariff *et al*., 2012, 2016). Unsedated TNE can be safely performed in a sitting position within a primary care office (Peery *et al*., 2012).

Several studies on the utility of TNE for screening for oesophageal cancer and precancerous conditions have been conducted. Previous RCTs demonstrated sensitivity for Barrett's oesophagus diagnosis ranging between 91% and 100%, and a sensitivity of 66.7–100% (Shariff *et al*., 2016), respectively. These results were sufficient to include unsedated TNE as an alternative approach for screening for this premalignant condition by the American College of Gastroenterology (Shaheen *et al*., 2016). Moreover, TNE may be a safe and well‐tolerable method for OSCC screening in high‐risk individuals. In previous studies including patients after head and neck and hypopharyngeal cancer, who are at risk for a second cancer, TNE has been shown to be a feasible and safe procedure with a performance of cancer detection comparable to chromoendoscopy with Lugol's staining (Arantes *et al*., 2013; Wang *et al*., 2014).

There are some downsides of TNE to consider. Investment is required in the hardware (some are portable and others not) and the operator needs to be a highly trained endoscopist. It provides a lower quality image and a smaller biopsy size, which may compromise the histopathological assessment (i.e. the portable version does not permit a biopsy to be taken). More studies on TNE technology in a larger set of individuals are required to understand the utility of this device as a screening modality for oesophagogastric cancer and premalignant conditions.

#### 2.1.3 Capsule endoscopy

The introduction of video capsule endoscopy (VCE) in the early 2000s provided a novel minimally‐invasive approach to evaluate the small bowel. VCE has become an important diagnostic tool in the management of obscure GI bleeding; however, its utility has been gradually expanding to include assessment of coeliac disease, small bowel tumours and hereditary polyposis syndromes. Recently, new models of endoscopic capsules adapted for the evaluation of the oesophagus (PillCam™ ESO; Given Imaging, Yokne'am Illit, Israel) and colon (PillCam™ COLON; Given Imaging) have been developed, opening a potential role for VCE as a screening modality.

Colon VCE might be particularly useful in screening patients who are unable to undergo colonoscopy (as an alternative to CT colonography). In a prospective multicentre trial in a cohort of patients after incomplete colonoscopy, VCE could identify more polyps than CT colonography and the complete colonic evaluation with VCE was achieved in 98% of cases (Spada *et al*., 2015).

More recently, a second‐generation colon VCE was developed and evaluated in a prospective study on 74 patients after incomplete colonoscopy. VCE detected significant polyps (size ≥ 6 mm or number ≥ 3) in 24% of cases. Importantly, most of the polyps (86%) were found in the right side of the colon, comprising segments that could not be visualised before as a result of incomplete endoscopy (Baltes *et al*., 2018).

VCE has also been investigated for the evaluation of oesophageal cancer and premalignant conditions. A meta‐analysis summarising nine studies on 618 patients with chronic GORD have shown that VCE could diagnose Barrett's oesophagus with a sensitivity of 77% and a specificity of 86%, respectively (Bhardwaj *et al*., 2009). Oesophageal VCE was found to be safe and had a high proportion of patient preferring this method over standard endoscopy (Bhardwaj *et al*., 2009). Its accuracy in OSCC detection, however, appears to be less encouraging. In a prospective cohort study on 68 patients at risk of OSCC secondary to a previous head and neck neoplasia, the per‐patient sensitivity, specificity, positive and negative predictive values were 63%, 86%, 77% and 76%, compared to conventional gastroscopy. These values have dropped down to 61%, 86%, 77% and 73%, respectively, compared to endoscopy with Lugol staining (Heresbach *et al*., 2010).

Further studies are needed to understand the utility of VCE as a screening tool for GI malignancy; however, high procedural costs and inability to take biopsies remain a limiting factor for this method.

### 2.2 Molecular biomarkers

#### 2.2.1 Stool tests for CRC detection

Molecular biomarkers are starting to play an important role in the detection and stratification of patients with GI malignancies aided by the rapid improvement and low cost of sequencing‐based technologies. For example, a biomarker test that has revolutionised CRC screening in the last decades is the faecal occult blood test (FOBT), which is currently the most widely used screening modality for this cancer (Zavoral *et al*., 2009). The two main types of FOBT include guaiac FOBT (gFOBT) and the FIT for haemoglobin. The former detects the pseudoperoxidase activity of the haemoglobin, whereas FIT detects the presence of globin by immunochemical reactions (Tinmouth *et al*., 2015). Although there are no controlled trials comparing FIT and gFOBT, some observational studies indicate that FIT increases the sensitivity for CRC detection (Allison *et al*., 1996; Brenner and Tao, 2013; Park *et al*., 2010; Parra‐Blanco *et al*., 2010). Moreover, a meta‐analysis reported better participation with FIT than gFOBT (relative risk = 1.16, 95% CI = 1.03–1.3) (Hassan *et al*., 2012).

FIT can detect both cancer and advanced adenomas (usually defined as size ≥ 10 mm and/or villous component > 20% and/or presence of high‐grade dysplasia) with a variable accuracy depending on the type of the test. According to a recent systematic review for the US Preventive Services Task Force, FIT in a single stool specimen has a sensitivity ranging between 73% and 88% and a specificity of 90–96% in CRC detection (Lin *et al*., 2016). The sensitivity in detecting advanced adenoma ranged from 22.2% to 40.3% (Chen *et al*., 2018).

New tests, such as the multitarget stool DNA test, combine both mutant and methylated DNA markers and a standard FIT. This test might even further increase the specificity for the detection of curable‐stage CRC and advanced adenomas, although this comes at a cost of slightly lower specificity (Imperiale *et al*., 2014).

#### 2.2.2 Non‐endoscopic cell collection devices coupled with *in vitro* tests for diagnosis of Barrett's and early oesophageal cancer

Given the costs associated with endoscopy‐based screening modalities, there is increasing interest in non‐endoscopic cell collection devices (Lao‐Sirieix *et al*., 2009; Moinova *et al*., 2018). This technology has been used previously in the context of OSCC screening but has failed as a result of a reliance on cytological assessment of atypia. The combination of an effective cell collection device coupled with biomarkers has proven more tractable for Barrett's oesophagus.

**Figure 2:**
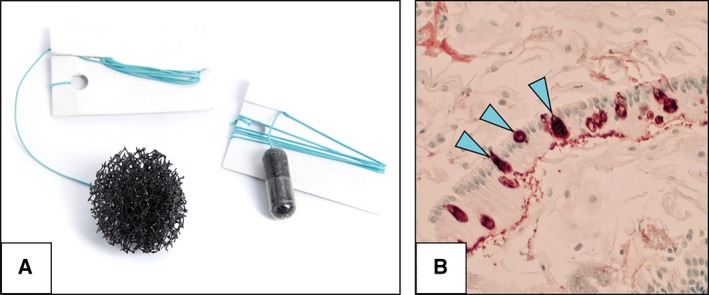
Cytosponge oesophageal cell collection device. (A) Cytosponge oesophageal cell collection device in a gelatin capsule (right) and expanded (left). (B) Trefoil‐factor 3 staining (20×) from a patient with Barrett's oesophagus showing columnar lined epithelium with goblet cells (arrowheads). Courtesy of Dr Maria O'Donovan (Department of Histopathology, Cambridge University Hospitals NHS Foundation Trust, Cambridge, UK).

Most data are available for Cytosponge (MRC Cancer Cell Unit, Cambridge, UK), which is a non‐endoscopic cell collection device that consists of a capsule attached to a string (Fig. 2A). After swallowing the device, the capsule coating disintegrates within 5 min upon reaching the stomach, revealing a 3‐cm spherical mesh that is withdrawn by pulling the string. Following its retrieval, the Cytosponge collects superficial cells from the length of the oesophagus. The device can be safely administered by a trained nurse in an office setting. The utility of the Cytosponge has been mainly focused on the early diagnosis of Barrett's metaplasia in the primary care setting; however, its potential role in OSCC screening is also being evaluated (OSCAR trial IRAS Project ID 155007).

Samples obtained from the Cytosponge can be assayed for various disease biomarkers, including the trefoil‐factor 3 (*TFF‐3*), which is highly specific to goblet cells, a histological landmark of Barrett's (Fig. 2B) (Lao‐Sirieix *et al*., 2009). This biomarker coupled with Cytosponge could diagnose this condition with a sensitivity of 79.9% (95% CI = 76.4–83.0%) and a specificity of 92.4% (95% CI = 89.5–94.7%) in a multicentre case–control study that included cases with an inadequate sample on an intention‐to‐treat basis (Ross‐Innes *et al*., 2015). The sensitivity increased to 87.2% (95% CI = 83.0–90.6%) for segments of Barrett's > 3 cm in length. Methylation or miRNAs are alternative biomarkers that could avoid the paraffin embedding and manual pathology review steps required for *TFF‐3* (Chettouh *et al*., 2017). Moreover, some potential biomarkers could stratify patients with Barrett's into low‐ and high‐risk groups for malignant progression using a combination of biomarkers including *TP53* (Moinova *et al*., 2018).

Screening with this device can be cost‐effective as shown in a recent microsimulation model. Cytosponge coupled with endoscopic therapy in a cohort of 50‐year‐old men with a history of GORD could provide a 19% reduction of incident OAC cases as compared to 17% for screening with endoscopy only. This benefit is mostly empowered by higher acceptability and uptake of the Cytosponge test compared to endoscopy (45% vs. 23%) (Benaglia *et al*., 2013). In another report, Cytosponge screening in GORD patients with a follow‐up endoscopic confirmation for positive cases would reduce the screening costs by 27–29% compared to endoscopic screening only (Heberle *et al*., 2017).

Importantly for implementation, Cytosponge is a safe and well‐tolerated procedure that can be widely deployed in primary care. In a recent systematic review of five prospective trials assessing its performance in 2418 patients with various oesophageal conditions, this test was associated with favourable acceptability of a median score of 6 points (IQR = 5.0–8.0) on the visual analogue scale (VAS). This score was higher compared to unsedated endoscopy (median 5.0, IQR = 3.0–7.0; *P* < 0.001). There were only two adverse events related to the device among all studies: a minor self‐limiting pharyngeal bleeding and one case of detachment (< 1 : 2000). Almost all patients successfully swallowed the Cytosponge (91.1%) (Januszewicz *et al*., 2018).

Taken together, Cytosponge coupled with *TFF‐3* is promising for a wide‐ranged screening; however, it still requires randomised trial data to fully evaluate its diagnostic yield, cost‐effectiveness and safety profile. This is currently underway in the BEST3 trial (ISRCTN68382401), which is a randomised trial in 13 000 individuals in multiple UK primary care sites (funded by Cancer Research UK) (Offman *et al*., 2018).

### 2.3 Serum biomarkers

In an ideal scenario, an early detection biomarker would be as non‐invasive as possible and inform for a variety of malignancies. Blood or breath sampling is feasible, although the challenge is to obtain specific sensitivity and specificity to abrogate false positives requiring extensive imaging or other work‐ups to find cancer. Progress is being made in this area.

#### 2.3.1 Serum pepsinogen, gastrin‐17 and *H. pylori* antibodies

A recent analysis from the USA showed that non‐invasive screening with serum pepsinogen may be a cost‐effective strategy to reduce gastric cancer mortality in a high‐risk population of actively smoking men aged > 50 years (Yeh *et al*., 2016). This test is aimed to identify individuals with atrophic gastritis and more advanced premalignant conditions, such as intestinal metaplasia and dysplasia. Serum pepsinogen and gastrin‐17 (G‐17) levels reflect the morphologic and functional status of the stomach mucosa. Pepsinogen I is secreted by chief and mucous neck cells in the fundic glands, whereas pepsinogen II is secreted by the cells in the pylorus. When atrophic changes develop in the corpus, the level of pepsinogen I decreases, and the level of pepsinogen II remains stable. Therefore, the pepsinogen I/II ratio changes in a stepwise manner and can be used to inform about the presence and grade of atrophic gastritis. Recent studies have shown that using a threshold of pepsinogen I level ≤ 70 μg·L^−1^ and a pepsinogen I/II ratio ≤ 3.0, this test could identify gastric atrophy (with and without more advanced precancerous lesions) with a sensitivity of 71% (95% CI = 59–82%) and specificity of 98% (95% CI = 97–99%) (Burucoa *et al*., 2013). The serological panel can be enriched with testing for *H. pylori* infection, which is the main driver for atrophic gastritis and a class I carcinogen according to the WHO (Vogiatzi *et al*., 2007). *Helicobacter pylori* serological testing and subsequent eradication of the bacteria could serve as an independent strategy for primary gastric cancer prevention.

A recent multicentre prospective cohort study from the Netherlands and Norway showed that the serological panel (pepsinogen and G‐17) might add additional value in stratifying patients with premalignant conditions of the stomach to those at higher and lower risk for malignant progression (den Hollander *et al*., 2018). Such stratification could lead to a reduction of unnecessary endoscopic surveillance in a large group of patients with a low‐risk profile. High‐quality data from randomised trials are needed to fully understand the accuracy and cost‐effectiveness of serum biomarker testing. An ongoing multicentre RCT of *H. pylori* eradication and pepsinogen testing for prevention of gastric cancer mortality (the GISTAR Study) might provide more information on the utility of this strategy (Leja *et al*., 2017).

#### 2.3.2 Circulating tumour DNA

The growing body of research on genetic alterations responsible for tumour formation and progression opened a new era of cancer detection and monitoring. For almost every cancer, a specific somatic mutation can be identified and potentially detected by the polymerase chain reaction‐based technologies. By contrast to well‐established serum protein biomarkers, such as the CEA or CA19‐9, mutations present in the circulating tumour DNA (ctDNA) appear to be more specific to the neoplastic tissue, which is advantageous for the accuracy of this method.

In a landmark study by Bettegowda *et al*. (2014) on 640 patients with cancers originating from 14 different tissue types, the specific ctDNA could be identified in 82% of solid tumours (outside the brain), although the concentration of ctDNA varied significantly among patients. Interestingly, CRC and gastro‐oesophageal cancers constituted a group with the highest fraction of detectable ctDNA (Bettegowda *et al*., 2014).

Not surprisingly, the proportion of patients with identifiable levels of ctDNA was increasing with the disease stages, as 47% of patients with stage I disease had detectable ctDNA, and 55%, 69%, and 82% with stages II, III, and IV, respectively. For colorectal and gastro‐oesophageal cancers, all patients with advanced disease had identifiable ctDNA and in 60–70% of patients with localised disease. The ctDNA concentration was also shown to have a prognostic role, as its increasing values were associated with decreasing survival rates (Bettegowda *et al*., 2014).

The utility of ctDNA was assessed in a separate cohort of 206 patients with metastatic CRC based on detecting mutations in the *KRAS* gene. This gene has a significant clinical value as it determines the possibility of treatment with EGFR‐inhibitors, such as panitumumab or cetuximab. The test identified 69 patients (33%) with detectable mutant *KRAS* genes in their plasma (out of 79 patients with *KRAS* mutation present in the tumour), which yielded a sensitivity of 87.2% with respect to cancer detection. Moreover, the study provided promising evidence that the ctDNA can also be used to detect a minimal residual disease after treatment and predicting an early relapse (Bettegowda *et al*., 2014).

In a more recent study, a novel test called CancerSEEK (a polymerase chain reaction‐based assay) was used to detect cell‐free ctDNA in a cohort of 1005 patients with non‐metastatic cancers of the ovary, liver, stomach, pancreas, oesophagus, colorectum, lung and breast (stages I to III) (Cohen *et al*., 2018). Notably, five of those cancers (i.e. ovary, liver, stomach, pancreas, and oesophagus) are lacking wide‐ranged screening tests for the average‐risk population. CancerSEEK could detect those five cancers with a sensitivity ranging from 69% to 98%. As before, the test showed increasing accuracy in detecting more advanced stages of the disease (sensitivity of 43%, 73% and 78% for stages I, II and III, respectively).

Most importantly, the CancerSEEK test could overcome the main limitation of ‘liquid‐biopsy’ technologies, which is an inability to identify the primary tumour site. This limitation arises from a fact that the same driver gene mutations can be shared by multiple cancer types. CancerSEEK used a combination of protein biomarkers and DNA mutations and, when coupled with a supervised machine learning module, could localise the source of cancer to two possible anatomic sites in a median of 83% of patients, and to a single organ in a median of 63% patients (Cohen *et al*., 2018). However, further validation in large prospective cohorts is required. This is especially important in view of recent data showing that typical driver mutations can occur in healthy cells throughout life, which does not necessarily alter the cell behaviour. For example, a study analysing the oesophageal epithelium from healthy donor samples showed the presence of *TP53* mutations in 5–10% across all nine donors, with the oldest donor (75 years old) having *TP53* mutations in 20–35% of cells (Martincorena *et al*., 2018). In another study, analysing the role of ctDNA in the early detection of small‐cell lung cancer, the *TP53* mutations were present in the plasma of 11% of the 225 non‐cancer controls (Fernandez‐Cuesta *et al*., 2016). Lastly, the *KRAS* cell‐free DNA mutations could be detected in 3.7% of healthy controls and in 4.3% of patients with chronic pancreatitis in a study focused on pancreatic cancer detection (Calvez‐Kelm *et al*., 2016).

To overcome those potential limitations, the ctDNA technology is constantly being refined. Recently, an immunoprecipitation‐based protocol was introduced, where further genotyping of ctDNA is being conducted to assess the tumour‐specific methylation patterns, which then can be detected in the plasma (Shen *et al*., 2018). This approach has the potential to improve the sensitivity and cost‐effectiveness of ctDNA technology; however, it is still on its very early phases of implementation and the field is evolving rapidly.

### 2.4 Breath tests

Electrical interfaces to measure the subtle volatile organic compounds (VOC) profiles of different diseases is a very attractive screening modality since it can be performed in a primary care setting. VOC is a carbon‐containing compound that can be detected in the gas phase at room temperature. Up to now, the established role of VOC measurements includes breathalysers for ethanol detection, carbon‐13 urea test for *H. pylori* infection and hydrogen‐methane testing for small‐bowel bacterial overgrowth. Recently, the analysis of VOCs within exhaled breath is being evaluated as a novel approach to the diagnosis of cancer.

A meta‐analysis of 63 studies on 3554 patients with different cancer types (mostly lung, breast and gastro‐oesophageal) has shown a sensitivity of breath testing for cancer diagnosis ranging from 28% to 100%, and a specificity of 61–100%. The overall pooled analysis showed a mean area under the receiver operating characteristic (ROC) curve of 0.9, and a pooled sensitivity and specificity of 79% (95% CI = 77–81%) and 89% (95% CI = 88–90%), respectively (Hanna *et al*., 2018). The substantial heterogeneity between the studies, including different methods of sample collection and test environment, remains a limitation of this analysis.

Moreover, breath testing appears to have a role in detecting premalignant disease. In a recent proof of concept cross‐sectional study using an e‐nose device to evaluate the breath VOCs in a cohort of patients with dysplastic Barrett's oesophagus (*n* = 122), the device showed a sensitivity of 82%, a specificity of 80% and an area under the curve of 0.79 (Chan *et al*., 2017) for the diagnosis of this condition. A 95% enrolment rate during the study indicated that this technique could be widely acceptable (Chan *et al*., 2017).

A number of different mass spectrometry methods are being evaluated which have the potential to dramatically improve the sensitivity. The ease of use means that breath tests detecting VOCs have a potential role in mass screening, however, standardisation and validation of this technique are required before implementation into clinical practice.

### 2.5 Minimally‐invasive treatment for early neoplasia

Over the last two decades, minimally‐invasive treatment methodologies have revolutionised the therapy of early neoplasia in the GI tract. Endoscopic resection techniques have shifted a large proportion of patients from invasive surgical treatment towards endoscopic therapy, which is characterised by higher patient tolerability, a minimal complication rate and similar, if not better, treatment outcomes. Moreover, endoscopic therapy plays a key role in the treatment of premalignant conditions, such as Barrett metaplasia or squamous dysplasia of the oesophagus as well as adenomas in the colon, making it one of the most important tools in preventive medicine. Below, we highlight the current and emerging minimally invasive treatment modalities for GI premalignant conditions and early cancers.

#### 2.5.1 Ablative treatment

Ablative therapies have been utilised with increasing frequency for the treatment of oesophageal premalignant conditions, such as dysplastic Barrett's oesophagus and squamous dysplasia. Several modalities are available for topical ablation, although argon plasma coagulation (APC) and radiofrequency ablation (RFA) remain the most commonly used. Ablative treatment is particularly indicated in cases of low‐grade dysplasia (LGD) or high‐grade dysplasia (HGD) without visible abnormality (‘flat dysplasia’) when a focal resection cannot be implemented. The aim of ablation is to eradicate (burn) the dysplastic area and allow re‐epithelialisation with normal mucosa.

Argon plasma coagulation involves the passage of argon gas through an endoscopic catheter with the conduction of monopolar current through the gas into the tissue. It is a relatively cheap and widely available ablation tool most commonly used to achieve haemostasis in bleeding vessels within the GI tract. However, the efficacy of APC in the treatment of dysplastic Barrett's has also been demonstrated. The evidence comes mainly from case series reports, and a recent RCT showing that APC could achieve a clearance of dysplasia in 83.8% cases and complete clearance of Barrett's metaplasia in 48.3% of the cases (Farhad *et al*., 2018). Recently, APC has been combined with a submucosal saline injection to improve its safety (hybrid‐APC) (Manner *et al*., 2016). The saline injection creates a submucosal cushion that can lower the risk of damaging the muscular layer of the oesophagus (decreasing the post‐procedural pain and risk of perforation) and, additionally, it allows the use of a higher energy setting, which improves the efficacy of ablation.

**Figure 3:**
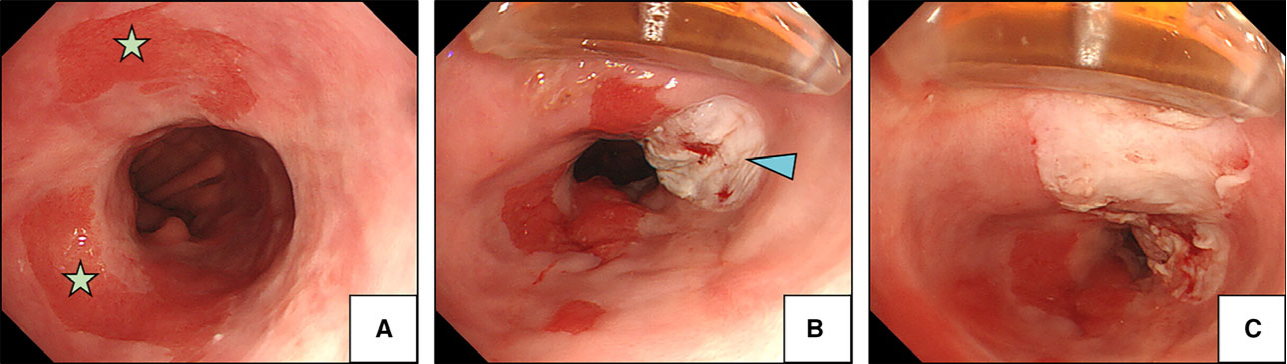
Radiofrequency ablation for Barrett's oesophagus. (A) Endoscopic image of the residual Barrett's oesophagus epithelium (asterisks) after previous endoscopic mucosal resection. (B) RFA90 device used for focal ablation of Barrett's epithelium. Arrowhead shows the area of single ablation with the RFA90 device. (C) Subsequent ablations are made to treat the whole remaining area of Barrett's epithelium. Patients provided written consent for the images to be used for educational purposes.

On the other hand, RFA is an accepted and most commonly used treatment modality for Barrett‐related neoplasia. This technique uses thermal energy to ablate the superficial layers of the oesophageal lining to a depth of approximately 1 mm. Two basic types of RFA include RFA360 and RFA90. RFA360 consists of a balloon with electrodes, which is insufflated within the oesophageal lumen to deliver a shallow circumferential burn. Differently, RFA90 is used to focally burn small areas of Barrett's epithelium (Fig. 3). In many countries, RFA has become the method of choice in the treatment of dysplastic Barrett's. In a landmark multicentre sham‐controlled trial by Shaheen *et al*. (2009), a complete eradication of Barrett's metaplasia was achieved in 77.4% of patients and this has resulted in lower disease progression rates (3.6% vs. 16.3%, *P* = 0.03) and fewer cancers (1.2% vs. 9.3%, *P *= 0.045) compared to the control group (sham procedure). Overall, the efficacy in achieving complete remission for Barrett's metaplasia after RFA treatment is ranging between 75–88% and remission of dysplasia between 88 and 92% (Haidry *et al*., 2013; Phoa *et al*., 2014; Shaheen *et al*., 2009). The most recent follow‐up study shows that the response to the RFA treatment can last up to 6 years (Klaver *et al*., 2018).

Some studies evaluated the efficacy of RFA in the treatment of oesophageal squamous dysplasia. Two prospective trials showed promising results with a complete remission achieved in 87% and 97% of the cases, respectively. However, the studies are limited by a small cohort of patients and a short follow‐up (< 12 months) (Bergman *et al*., 2011; He *et al*., 2015).

An emerging concept in the treatment of oesophageal premalignant disease is cryoablation. Although this method has been used for decades in the treatment of precancerous dermatologic and gynecologic conditions, it has only been recently introduced for treatment within the oesophagus. Cryoablation uses liquid nitrogen at a temperature of −196 °C that is topically applied through a low‐pressure spray to the oesophageal mucosa (Gosain *et al*., 2013). A newer modification of this system employs a balloon that is inflated in the oesophageal lumen and a nitrous oxide gas spray is used to freeze target mucosa that is being in contact with the balloon. In a prospective study with 41 patients with Barrett's, a complete eradication of dysplasia was achieved in 95% of individuals and complete eradication of intestinal metaplasia in 88% of patients, respectively, at 1 year after the procedure (Canto *et al*., 2018). Cryoablation is a promising tool in the treatment of premalignant conditions in the oesophagus; however, larger randomised studies are needed to fully understand its efficacy and safety.

#### 2.5.2 Resection techniques

GI cancers at an early stage of disease, with a low risk of lymph node metastases or distant spread, can be managed endoscopically with comparable long‐term survival rates to surgery. This includes lesions limited to the mucosa and the superficial layers of the submucosa, which are the most amenable to endoscopic cure. The two main resection techniques include endoscopic mucosal resection (EMR) and submucosal dissection (ESD). Indications for both EMR and ESD are constantly expanding and generally include focal dysplastic lesions in the oesophagus and the stomach, early oesophageal cancers (T1a), early gastric cancers (T1a), colonic polyps and early colorectal neoplasia.

Endoscopic mucosal resection offers both diagnostic and therapeutic capability. It is typically used to resect neoplastic lesions of less than 2 cm in size, or larger, in a piece‐meal fashion. There are several EMR techniques. In injection‐assisted EMR, the target lesion is lifted with a submucosal injection of a fluid (typically saline with epinephrine and indigo carmine dye) and then resected with a cautery snare, comprising the most commonly used method for the treatment of polypoid lesions in the colon. In the oesophagus and the stomach, however, a cap‐assisted EMR is preferred. This technique uses a transparent suction cap that is placed on the tip of the endoscope, with a pre‐opened snare at its distal edge. The target lesion is suctioned into the cap and subsequently cut‐off with the cautery snare. Alternatively, a ligation EMR technique can be used, where a suction cap is equipped with rubber bands that are deployed after suctioning the target tissue into the cap. This creates a pseudo‐polyp with the neoplastic tissue included, which is then resected with the snare beneath the base of the band.

Endoscopic mucosal resection provides histological information including important prognostic factors such as the degree of cancer differentiation, presence or absence of lymphovascular invasion, depth of cancer invasion, and the distance of cancer from the deep and lateral resection margins. These criteria determine whether the curative intent of endoscopic treatment was achieved. Lesions confined to the mucosa have a very low rate of lymphatic involvement; therefore, EMR is considered curative for most GI cancers limited to the mucosal layer (T1a) in conjunction with low or moderate differentiation of cancer, no lymphovascular invasion and clear resection margins.

In some cancers, such as OAC, there is emerging evidence that even with a presence of superficial submucosal invasion (less than 500 μm; T1b‐Sm1), with a good or moderate differentiation (G1/G2), no lymphovascular invasion and clear resection margins, can be safely managed endoscopically because a growing body of evidence supports a low risk of nodal spread in this stage.

By contrast, the risk of nodal spread in OSCC is much higher than in OAC and only cancers limited to the top layers of the mucosa (m1 and m2) are considered safe in terms of endoscopic treatment (Cho *et al*., 2014). The risk of nodal metastases in lesion penetrating into the deep mucosal layer (m3) and superficial submucosal layers (Sm1) can be as high as 18% and 50%, respectively, according to some stusies (Cho *et al*., 2014).

**Figure 4:**
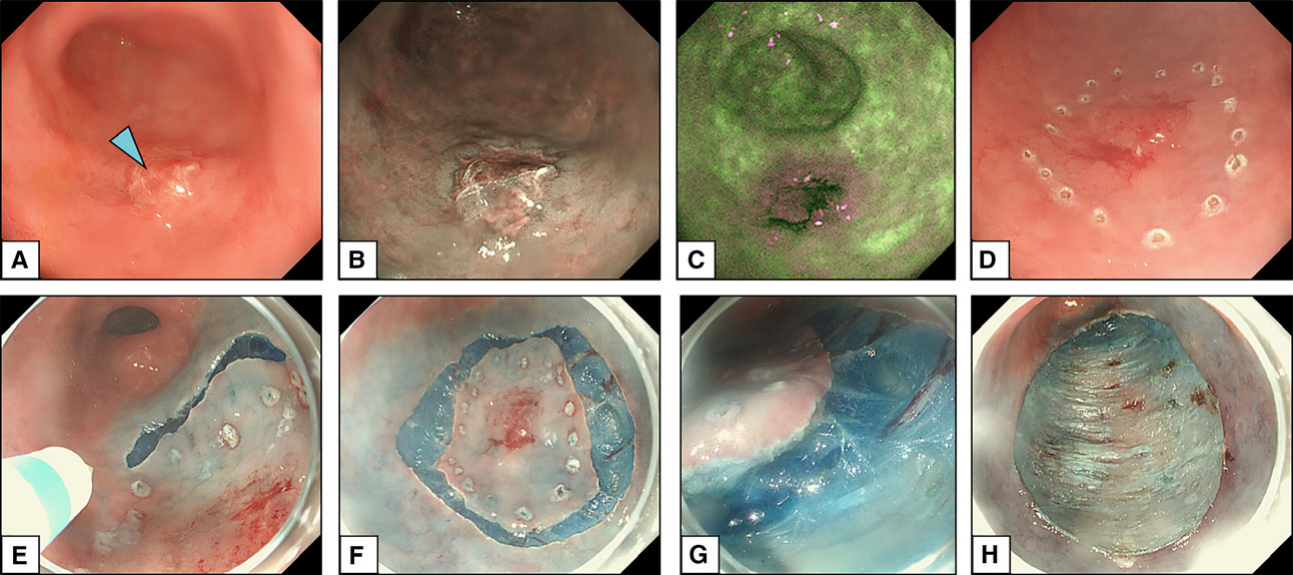
Endoscopic submucosal dissection for early gastric cancer. Courtesy of Dr Massimiliano di Pietro (MRC Cancer Unit, University of Cambridge, Cambridge, UK). (A) Endoscopic image of an early gastric cancer seen in white light imaging (arrowhead). (B, C) Advanced imaging techniques, such as narrow‐band imaging (B) and autofluorescence imaging (C) help to delineate the borders of the lesion. (D) Marking around the lateral margins of the lesion using the tip of the endoscopic knife. (E, F) Circumferential cutting around the margins of the lesion. (G, H) Dissection and removal of the whole specimen revealing the muscle layer (muscularis propria) of the gastric wall. Patients provided written consent for the images to be used for educational purposes.

Endoscopic submucosal dissection (ESD) permits en‐bloc resection of larger lesions than with EMR. Initially developed for gastric tumours, the utility of ESD has expanded to include the treatment of oesophageal and colorectal neoplasia. This procedure typically consists of several steps, including delineation and marking around the lateral margins of the lesion, injection of fluid underneath the target tissue, circumferential cutting, dissection of the submucosal layer, coagulation of visible vessels, and removal of the resected specimen (Fig. 4). Currently, ESD is the method of choice in the treatment of early gastric cancers and OSCC. For oesophageal adenocarcinomas and early colorectal cancers, EMR still remains the most commonly used technique in the Western countries; however, ESD may be considered in selected cases, such as for larger lesions (>2 cm), poorly lifting lesions and lesions at increased risk for submucosal invasion.

## 3 Conclusions

In recent decades, substantial progress has been made in the field of early cancer detection and therapy. This advancement can be readily appreciated for GI malignancies, comprising one of the most prevalent group of cancers globally. We are witnessing an increasing trend where well‐established invasive screening modalities, such as endoscopic screening, are being increasingly replaced by less invasive and biomarker‐driven tests. Endoscopic therapy, on the other hand, has become the main treatment modality not only for early cancers, but also for premalignant conditions of the GI tract. Taken together, there is a real opportunity to cause a significant shift in the stage of GI cancer diagnosis with an impact on population mortality in the longer term, as well as a reduction in the morbidity associated with cytotoxic‐ and surgical‐based treatments and expensive molecular targeted agents in precision medicine. With ever increasing improvements in early detection methods, we can expect this paradigm to be increasingly applied for a variety of cancers.

## Author contributions

Both authors contributed to the writing of this manuscript.

## Conflict of interest

Rebecca C. Fitzgerald is a named inventor on patents pertaining to the Cytosponge.
